# Supplementation of *Citrus maxima* Peel Powder Prevented Oxidative Stress, Fibrosis, and Hepatic Damage in Carbon Tetrachloride (CCl_4_) Treated Rats

**DOI:** 10.1155/2015/598179

**Published:** 2015-05-28

**Authors:** Mohammed Riaz Hasan Chowdhury, Md Abu Taher Sagor, Nabila Tabassum, Md Abdullah Potol, Hemayet Hossain, Md Ashraful Alam

**Affiliations:** ^1^Department of Pharmaceutical Sciences, North South University, Bashundhara, Dhaka 1229, Bangladesh; ^2^BCSIR Laboratories, Bangladesh Council of Scientific and Industrial Research (BCSIR), Dhaka 1205, Bangladesh

## Abstract

*Citrus maxima* peel is rich in natural phenolic compounds and has a long use in the traditional medicine. HPLC-DAD analysis on *Citrus maxima* peel powder exhibited the presence of various phenolic compounds such as caffeic acid and (−)-epicatechin. To determine the plausible hepatoprotective activity of *Citrus maxima* peel powder, we used carbon tetrachloride (CCl_4_) treated rat model. Liver damage in rats was confirmed by measuring the AST, ALT, and ALP enzyme activities. In addition, lipid peroxidation products (MDA), nitric oxide, advanced protein oxidation products level (APOP), and catalase activities were also analyzed along with the histological profiling for the inflammatory cell infiltration, collagen, and iron deposition in liver. Dietary supplementation of *Citrus maxima* peel powder exhibited significant reduction of serum AST, ALT, and ALP activities in carbon tetrachloride treated rats. Moreover, *Citrus maxima* peel powder also showed a significant reduction of the oxidative stress markers (MDA, NO, and APOP level) and restored the catalase activity in CCl_4_ treated rats. Histological examination of the liver section revealed reduced inflammatory cells infiltration, collagen, and iron deposition in CCl_4_ treated rats. The results from this study demonstrated that *Citrus maxima* peel powder produced significant hepatoprotective action in CCl_4_ administered rats.

## 1. Introduction

Chronic liver diseases have become a public health concern due to their high morbidity and mortality rates. In the recent years, fibrosis in the liver and liver damage have increased drastically [1]. Alcoholic and nonalcoholic liver diseases are predisposed by oxidative stress and inflammation which may further trigger the fibrosis in the liver [1–3]. Progression of the inflammatory diseases also involves various proinflammatory mediators such as interleukins, cytokines, and nuclear factor-*κ*B (NF-*κ*B) [4]. Generally in oxidative stress condition Kupffer cells play a crucial role in the progression of liver damage by releasing more cytokines, such as platelet-derived growth factor (PDGF), transforming growth factor-1 (TGF-1), tumor necrosis factor-*α* (TNF-*α*), and endothelin-1 (ET-1) [5]. Chronic inflammation due to oxidative stress and inflammation also activates the hepatic stellate cells (HSCs), which produce extracellular matrix (ECM) deposition and fibrosis in liver [5]. Carbon tetrachloride (CCl_4_) is a well-known hepatotoxin that has been widely used to induce hepatic injuries in experimental animal [6]. CCl_4_ induces oxidative stress and massive inflammatory cell infiltration in rat's liver, which mimics symptoms of various human liver dysfunctions [7]. Conventional drugs used in the treatment of liver diseases are still inadequate and many have doubtful efficacy and safety [8]. This warrants an extensive search for the alternative drugs for the treatment of liver disease is necessary.

Citrus plants belong to the Rutaceae family and genus* Citrus*. They are native to Southeast Asian countries like Bangladesh, India, and China. Their peels and seeds are rich source of phenolic compounds, which include phenolic acids and flavonoids [9]. Citrus flavonoids have various therapeutic properties, which include anticancer, antiviral and anti-inflammatory activities, and an ability to inhibit human platelet aggregation [9]. Hesperidin and naringin are beneficial for improving hyperlipidemia and hyperglycemia in type 2 diabetic animals by partly regulating the fatty acid and cholesterol metabolism affecting the gene expression of glucose regulating enzymes [10]. Our previous study also demonstrated that naringin may prevent the hypertension and obesity related cardiovascular complications in high fat high carbohydrate diet fed rats [11]. Moreover, naringin prevented hepatic steatosis, inflammation and fibrosis by ameliorating oxidative stress in diet induced obese rats [11]. Many types of citrus fruits can be found around the globe. However,* Citrus maxima* (J. Burm.) Merr. (Rutaceae) receives attention for its large size fruits.* Citrus maxima* fruit is known as pomelo in English and batabi lebu in Bengali, which is widely grown throughout Bangladesh, India, and East Asia.* Citrus maxima* fruits have been used for many diseases in traditional medicine. In the traditional medicine the pulp of* Citrus maxima* fruit is said to possess appetizing, antitoxic, cardiac stimulant, and stomach tonic properties [12].* Citrus maxima* fruit juice also possesses high amount of polyphenolic compounds like hesperidin, naringin, caffeic acid,* p*-coumaric acid, ferulic acid, and vanillic acid [13]. In our extensive search for any biochemical work done on* Citrus maxima*, we found that no experiment has been undertaken to elucidate the hepatoprotective and antifibrotic activity of* Citrus maxima* in CCl_4_ treated rats. Therefore, our current study was conducted to unveil the potential therapeutic effect of* Citrus maxima* peel powder in liver diseases.

## 2. Material and Methods

### 2.1. Chemicals

Arbutin (AR), gallic acid (GA), hydroquinone (HQ), (+)-catechin hydrate (CH), vanillic acid (VA), caffeic acid (CA), syringic acid (SA), (−)-epicatechin (EC), vanillin (VL),* p*-coumaric acid (PCA),* trans*-ferulic acid (FA), rutin hydrate (RH), ellagic acid (EA), benzoic acid (BA), rosmarinic acid (RA), myricetin (MC), quercetin (QU),* trans*-cinnamic acid (TCA), and kaempferol (KF) were purchased from Sigma-Aldrich (St. Louis, MO, USA). Acetonitrile (HPLC), methanol (HPLC), acetic acid (HPLC), and ethanol were obtained from Merck (Darmstadt, Germany).

### 2.2. Plant Sample Collection and Identification

*Citrus maxima* fruits were collected from the local market and the samples were identified by the expert Mr. Sarker Nasir Uddin, Senior Scientific Officer, National Herbarium, Mirpur, Dhaka, Bangladesh. A voucher specimen (acc. number 40844) was deposited in National Herbarium, Dhaka, Bangladesh, for future reference.

### 2.3. HPLC-DAD Analysis for Phenolic Compound in Ethanol Extract of* C. maxima* Peel Powder

Detection and quantification of phenolic compounds in the ethanol extract were determined by HPLC-DAD analysis as described elsewhere with some modifications [14, 15]. It was carried out on a Dionex UltiMate 3000 system equipped with quaternary rapid separation pump (LPG-3400RS) and photodiode array detector (DAD-3000RS). Separation was performed using Acclaim C_18_ (5 *μ*m) Dionex column (4.6 × 250 mm) at 30°C with a flow rate of 1 mL/min and an injection volume of 20 *μ*L. The mobile phase consisted of acetonitrile (solvent A), acetic acid solution pH 3.0 (solvent B), and methanol (solvent C) with the gradient elution program of 5%A/95%B (0–5 min), 10%A/90%B (6–9), 15%A/75%B/10%C (11–15), 20%A/65%B/15%C (16–19 min), 30%A/50%B/20%C (20–29 min), 40%A/30%B/30%C (30–35), and 100%A (36–40 min). The UV detector was set to 280 nm for 22.0 min, changed to 320 nm for 28.0 min, changed again to 280 nm for 35 min, and finally changed to 380 nm for 36 min and held for the rest of the analysis period, while the diode array detector was set at an acquisition range from 200 nm to 700 nm. For the preparation of calibration curve, a standard stock solution was prepared in methanol containing arbutin (AR), (−)-epicatechin (ECA) (5 *μ*g/mL each), gallic acid (GA), hydroquinone (HQ), vanillic acid (VA), rosmarinic acid (RA), myricetin (MC) (4 *μ*g/mL each), caffeic acid (CA), syringic acid (SA), vanillin (VL),* trans*-ferulic acid (FA) (3 *μ*g/mL each),* p*-coumaric acid (PCA), quercetin (QU), kaempferol (KF) (2 *μ*g/mL each), (+)-catechin hydrate (CH), ellagic acid (EA) (10 *μ*g/mL each),* trans*-cinnamic acid (TCA) (1 *μ*g/mL), rutin hydrate (RH) (6 *μ*g/mL), and benzoic acid (BA) (8 *μ*g/mL). A solution of the extract was prepared in ethanol having the concentration of 10 mg/mL. Prior to HPLC analysis, all the solutions (mixed standards, sample, and spiked solutions) were filtered through 0.20 *μ*m syringe filter (Sartorius, Germany) and then degassed in an ultrasonic bath (Hwashin, Korea) for 15 min. Data acquisition, peak integration, and calibrations were calculated with Dionex Chromeleon software (version 6.80 RS 10).

### 2.4. Animals and Treatment

24 ten- to twelve-week-old, Long-Evans female rats (150–180 g) were obtained from Animal Production Unit of Animal House at the Department of Pharmaceutical Sciences, North South University, and were kept in individual cages at room temperature of 25 ± 3°C with a 12 h dark/light cycles. They have free access to standard laboratory feed (pellet food crushed to coarse powder) and water, according to the study protocol approved by Ethical Committee of Department of Pharmaceutical Sciences, North South University, for animal care and experimentation. To study the hepatoprotective effects of* Citrus maxima*, rats were divided into four groups (5–7 rats in each group) named as Control (group I), Control +* Citrus maxima* (group II), CCl_4_ (group III), and CCl_4_ +* Citrus maxima* (group IV). Animals of group I were treated with 1 mL/kg of saline (0.85%) and olive oil (1 mL/kg) intragastrically twice a week for two weeks. Rats of groups III and IV were treated with CCl_4_ (1 : 3 in olive oil) at a dose of 1 mL/kg intragastrically twice a week for two weeks. However, animals of groups II and IV received* Citrus maxima* fruits peel powder in crashed powder of pellet food (0.5% of powder food, w/w) for two weeks. Animals were checked for the body weight and food and water intake on a daily basis. After 14 days, all animals were weighted and sacrificed and the blood and all internal organs such as heart, kidney, spleen, and liver were collected. Immediately after collection of the organs, they are weighted and stored in neutral buffered formalin (pH 7.4) for histological analysis and in refrigerator at −20°C for further analysis. Collected blood samples were centrifuged at 8000 rpm and the plasma was separated and stored in refrigerator at −20°C for further analysis.

### 2.5. Assessment of Hepatotoxicity

Liver marker enzymes (alanine aminotransferase (ALT), aspartate aminotransferase (AST), and alkaline phosphatase (ALP)) were estimated in plasma by using Diatech diagnostic kits (Hungary) according to the manufacturer's protocol.

### 2.6. Preparation of Tissue Sample for the Assessment of Oxidative Stress Markers

For determination of oxidative stress markers, liver tissue was homogenized in 10 volumes of phosphate buffer containing pH 7.4 and centrifuged at 12,000 ×g for 30 min at 4°C. The supernatant was collected and used for the determination of protein and enzymatic studies as described below.

### 2.7. Estimation of Lipid Peroxidation Concentration

Lipid peroxidation in liver was estimated colorimetrically measuring malondialdehyde (MDA) followed by previously described method [16]. In brief, 0.1 mL of tissue homogenate (Tris-Hcl buffer, pH 7.5) was treated with 2 mL of (1 : 1 : 1 ratio) TBA-TCA-HCl reagent (thiobarbituric acid 0.37%, 0.25 N HCl, and 15% TCA) and placed in hot water bath for 15 min and cooled. The absorbance of clear supernatant was measured against reference blank at 535 nm.

### 2.8. Estimation of Nitric Oxide (NO) Concentration

Nitric oxide (NO) was determined according to the method described by Tracey et al. as nitrate [17]. In this study, Griess-Ilosvay reagent was modified by using naphthyl ethylenediamine dihydrochloride (0.1% w/v) instead of 1-napthylamine (5%). The reaction mixture (3 mL) containing brain homogenates (2 mL) and phosphate buffer saline (0.5 mL) was incubated at 25°C for 150 min. A pink colored chromophore was formed which was measured at 540 nm.

### 2.9. Estimation of Advanced Oxidation Protein Products (APOP) Concentration

Determination of APOP levels was performed by modification of the method of Witko-Sarsat et al. [18] and Tiwari et al. [19]. 2 mL of plasma was diluted 1 : 5 in PBS: 0.1 mL of 1.16 mM potassium iodide was then added to each tube, followed by 0.2 mL acetic acid after 2 min. The absorbance of the reaction mixture was immediately read at 340 nm against a blank containing 2 mL of PBS, 0.1 mL of KI, and 0.2 mL of acetic acid. The chloramine-T absorbance at 340 nm was found linear within the range of 0 to 100 nmol/mL; APOP concentrations were expressed as nmol·mL^−1^ chloramine-T equivalents.

### 2.10. Estimation of Catalase Activity (CAT)

CAT activities were determined using previously described method by Chance and Maehly [20, 21] with some modifications. The reaction solution of CAT activities contained 2.5 mL of 50 mmol phosphate buffer (pH 5.0), 0.4 mL of 5.9 mmol H_2_O_2_, and 0.1 mL enzyme extract. Changes in absorbance of the reaction solution at 240 nm were determined after one minute. One unit of CAT activity was defined as an absorbance change of 0.01 as units/min.

### 2.11. Estimation of Reduced Glutathione (GSH) Concentration

Reduced glutathione was estimated by previously described method [22]. The total volume of 3.0 mL assay mixture was composed of 0.1 mL filtered aliquot, 2.7 mL phosphate buffer (0.1 M, pH 7.4), and 0.2 mL DTNB (5,5-dithiobis-2-nitrobenzoic acid) (100 mM). The yellow color of the mixture was developed, read immediately at 412 nm on a SmartSpec Plus Spectrophotometer, and expressed as ng/mg protein.

### 2.12. Estimation of Total Protein Concentration

Total protein in samples was determined by BCA protein acid kit (Thermo Scientific) according to the manufacturer's protocol.

### 2.13. Histopathological Determination

For microscopic evaluation, liver tissues were fixed in neutral buffered formalin and embedded in paraffin, sectioned at 5 *μ*m, and subsequently stained with hematoxylin/eosin to see the architecture of hepatic tissue and inflammatory cell infiltration. Sirius red staining for fibrosis and Prussian blue staining for iron deposition were also done in liver sections. Sections were then studied and photographed under light microscope (Zeiss Axio Scope) at a magnification of 40x.

### 2.14. Statistical Analysis

All values are expressed as mean ± standard error of mean (SEM). The results were evaluated by using the one-way ANOVA followed by Bonferroni or Newman-Keuls test using GraphPad Prism Software, version 6. Statistical significance was considered when *p* < 0.05 in all cases.

## 3. Results

### 3.1. HPLC-DAD Analysis for Phenolic Compound in Ethanol Extract of* C. maxima* Peel Powder

**Figure 1:**
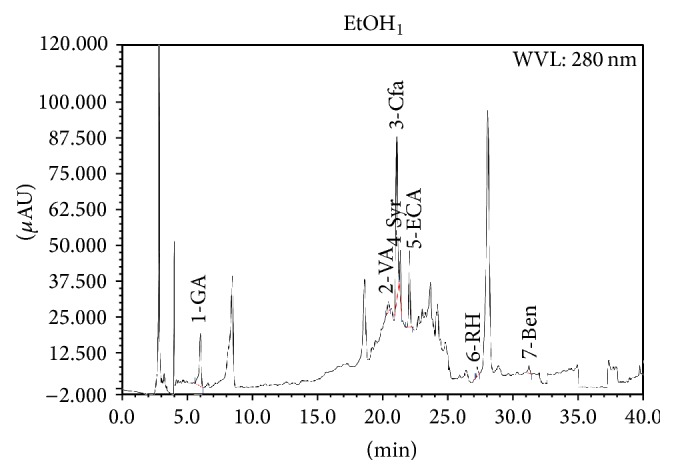
HPLC chromatogram of* Citrus maxima* peel extract. Peaks: 1, gallic acid; 2, vanillic acid; 3, caffeic acid; 4, syringic acid; 5, (−)-epicatechin; 6, rutin hydrate; 7, benzoic acid.

Figure 1 shows the chromatogram for the ethanol extract of* Citrus maxima* peel powder. A sharp peak of caffeic acid and (−)-epicatechin were seen in the chromatogram (concentration of 240.78 and 242.19 mg per 100 g of dry weight, resp., Table 1). The described conditions also have been suitable for the separation of the ethanol extract for which the chromatogram peak of other phenolic compounds like gallic acid, vanillic acid, syringic acid, rutin hydrate, and benzoic acid were also noticed. However, naringin and hesperidin were not analyzed due to unavailability of these compounds in our laboratory. Both caffeic acid and (−)-epicatechin are potent antioxidants which may have been contributed to the antioxidant activity of the extracts of* Citrus maxima* peel powder.

### 3.2. Effect on Body Weight, Food and Water Intake, and Organ Wet Weight

**Figure 2:**
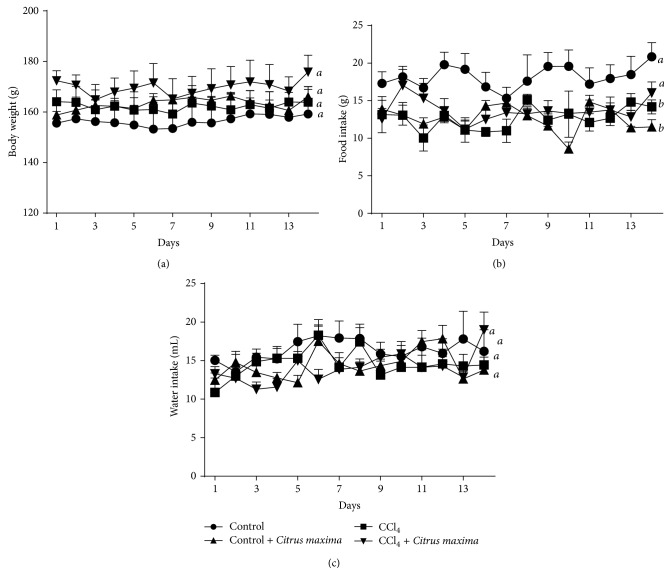
Effect of* Citrus maxima* peel powder on body weight (a), food intake (b), and water intake (c) in CCl_4_ treated rats.

Body weight of each rat was recorded every day during the experiment, and % change was calculated for all groups. It was found that the body weight did not decrease significantly in CCl_4_-intoxicated rat group compared to the Control rats. On the other hand, treatment of CCl_4_-intoxicated group with* Citrus maxima* showed no effect on the weight of rats (Figure 2). CCl_4_-intoxicated group significantly decreased food and water intake compared to Control rats; reduction of food and water intake was not improved by* Citrus maxima* peel powder in CCl_4_-intoxicated group (Figure 2).

Table 2 shows the effect of various treatments on the rats' organs wet weight. The spleen wet weight increased significantly (*p* < 0.05) in the CCl_4_ treated rats when compared with Control.* Citrus maxima* (0.5% powder food) treatment significantly (*p* < 0.05) attenuated the wet weight of the spleen in the CCl_4_ treated rats. CCl_4_ treated rats also showed slight decrease in liver wet weight; however,* Citrus maxima* (0.5% powder food) treatment did not change the wet weight of the liver compared to the Control. Another crucial finding in this study was the reduction of kidney wet weight and increment of the heart wet weight due to CCl_4_ intoxication.

### 3.3. Effect on Biochemical Parameter of Liver Functions

Biochemical measurement of liver functions revealed that CCl_4_ induced a significant increase in plasma AST, ALT, and ALP activity compared with Control values, respectively (Table 3). Treatment of animals with* Citrus maxima* (0.5% powder food) concomitantly with CCl_4_ significantly counteracted the alteration in all hepatotoxicity indices compared to the CCl_4_-intoxicated group. In addition, treatment of animals with* Citrus maxima* alone for 2 weeks did not show any significant change in liver enzymes compared with the Control group (Table 3).

### 3.4. Oxidative Stress Markers and Antioxidant Enzymes

To determine the oxidative stress in our study, we evaluated the MDA, nitric oxide, and APOP content in plasma and liver homogenates. CCl_4_ induced rats showed a higher concentration of lipid peroxidation product (MDA) both in plasma and liver homogenates (Table 3) (7.7 ± 0.91 and 57.16 ± 6.44 nmol/mL in plasma and liver homogenates, resp.). Additionally,* Citrus maxima* (0.5% of powder food) cotreatment significantly reduced the level of lipid peroxides compared to CCl_4_-intoxicated group (6.71 ± 0.77 and 36.92 ± 1.98 nmol/mL in plasma and liver homogenates, resp.); however, lipid peroxide level was still higher than the Control.

CCl_4_ has profound effect on APOP development in plasma and liver. CCl_4_ challenge significantly increased the APOP concentration in plasma and liver (164.4 ± 15.15 and 1357.62 ± 91.06 nmol/mL equivalent to chloramine-T, resp.) which was decreased due to* Citrus maxima* (0.5% of powder food) supplementation in CCl_4_-intoxicated rats (84.3 ± 8.8 and 977.50 ± 63.05 nmol/mL equivalent to chloramine-T, resp.) (Table 3).

Nitric oxide measured as nitrate was also increased in both plasma and liver homogenates (5.8 ± 0.3 and 35.62 ± 4.21 nmol/mL in plasma and liver homogenates, resp.) compared to Control rats which was normalized by* Citrus maxima* peel powder supplementation in CCl_4_-intoxicated group (Table 3). CCl_4_ induced a significant decrease in liver antioxidant enzyme GSH concentration and CAT activities, respectively, compared to the Control rats. In addition, CCl_4_ induced a significant, almost twofold increase in lipid peroxide level compared with the Control group (Table 3). Treatment of animals with activities (0.5% of powder food) concomitantly with CCl_4_ significantly counteracted the oxidative stress effect of CCl_4_. It was found that the GSH concentration and CAT activities were restored to near normal compared to CCl_4_-intoxicated group by* Citrus maxima* peel powder supplementation (Table 3).

### 3.5. Inflammation, Fibrosis Iron Deposition in Liver

**Figure 3:**
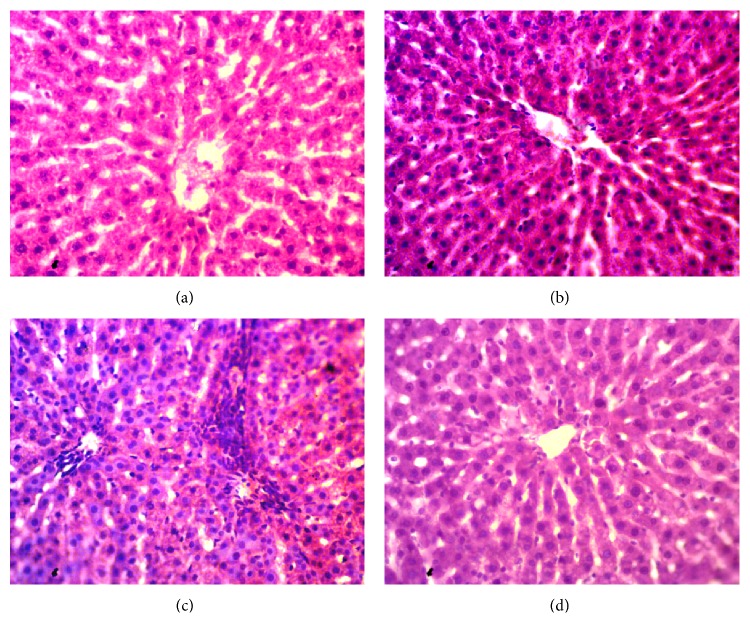
Effect of* Citrus maxima* peel powder on hepatic inflammation in CCl_4_ treated rats. Magnification 40x. (a) Control. (b) Control +* Citrus maxima*. (c) CCl_4_. (d) CCl_4_ +* Citrus maxima*.

**Figure 4:**
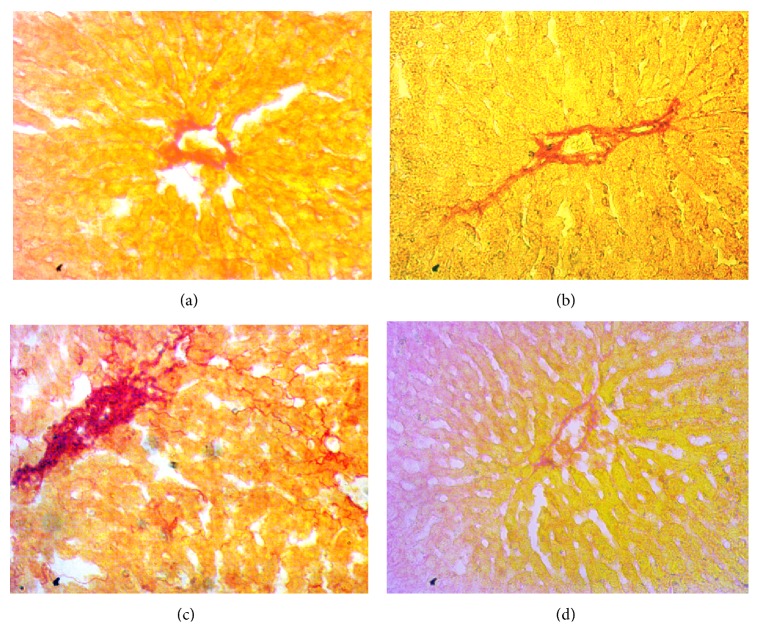
Effect of* Citrus maxima* peel powder on hepatic fibrosis in CCl_4_ treated rats. Magnification 40x. (a) Control. (b) Control +* Citrus maxima*. (c) CCl_4_. (d) CCl_4_ +* Citrus maxima*.

**Figure 5:**
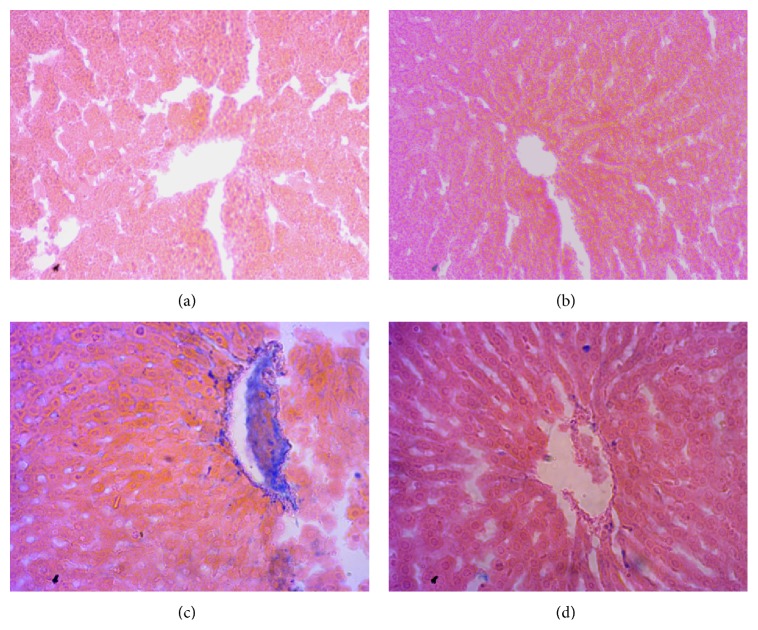
Effect of* Citrus maxima* peel powder on hepatic iron deposition in CCl_4_ treated rats. Magnification 40x. (a) Control. (b) Control +* Citrus maxima*. (c) CCl_4_. (d) CCl_4_ +* Citrus maxima*.

Inflammation was seen in rats treated with CCl_4_. Massive serge of inflammatory cells was found in the centrilobular part of liver section stained with H&E staining in CCl_4_ treated rats group (Figure 3(c)). Necrotized tissue scar and ballooning of the hepatocytes were also seen in liver of CCl_4_ treated rats.* Citrus maxima* (0.5% of powder food) cotreatment attenuated the inflammatory cell infiltration and necrosis in the liver tissues of CCl_4_ treated rats (Figure 3(d)). Liver fibrosis was evaluated histologically by visualizing the red color of collagen fibers using Sirius red stain. In contrast, the collagen fibers were heavily deposited around portal tracts and central veins in CCl_4_-intoxicated group and extended from central vein to portal tract resulting in the formation of pseudolobules (Figure 4(c)).* Citrus maxima* (0.5% of powder food) supplementation prevented the collagen deposition and fibrosis in CCl_4_-intoxicated rats (Figure 4(d)). Histological staining also revealed massive iron deposition in liver section stained for free iron deposition in CCl_4_ treated rats (Figure 5(c)).* Citrus maxima* supplementation decreased this iron deposition in CCl_4_ treated rats (Figure 5(d)).

## 4. Discussion

Liver damage in most cases involves oxidative stress and is characterized by progressive evolution from steatosis to chronic hepatitis, fibrosis, cirrhosis, and hepatocellular carcinoma. It is also the major target organ of chemical-induced toxicity. CCl_4_ is a hepatotoxin widely used in animal models for liver diseases. Several studies suggested that CCl_4_ produces toxicity by producing highly lethal trichloromethyl radical (^•^CCl_3_) and peroxy trichloromethyl free radical (^•^OOCCl_3_) through the activation of drug-metabolizing enzymes located in the endoplasmic reticulum [23], causing oxidative damage to cellular structure and macromolecules. The present study was conducted to evaluate the protective effect of* Citrus maxima* peel powder against CCl_4_ induced hepatic disorders in rats. Our results suggest that* Citrus maxima* peel powder possesses protective action against hepatic damages induced by CCl_4_.

CCl_4_ is a well-known hepatotoxic agent which is metabolized through hepatic cytochrome P_450_ enzymes and produces excessive free radicals [24]. Free radicals are known to cause oxidative damage to a number of molecules in the cell, including membrane lipids, proteins, and nucleic acids. The increased MDA levels in the liver of CCl_4_ treated animals indicate tissue injury caused due to lipid peroxidation. Previous studies suggest that oxidative stress marker enzymes such as catalase activity and GSH concentrations are lowered by free radicals resulting in liver damage [25]. The results of the present study also revealed that CCl_4_ administration caused a significant elevation of serum marker enzymes activities such as ALT, AST, and ALP. Elevation of AST and ALT activity is a marker of severe acute liver damage in rats as demonstrated by CCl_4_ administration [25, 26]. These enzyme activities were significantly normalized by* Citrus maxima* peel powder supplementation. Furthermore, the significant depletion of MDA concentration in plasma and liver tissue of the* Citrus maxima* treated animals might be due to decreased lipid peroxidation or enhanced tissue antioxidant defense enzyme activities.

Our study also suggests that CCl_4_ administration causes inflammatory cell infiltration in liver of rats. Generally, Kupffer cells (local macrophage type cells in liver) could activate hepatic stellate cells by various inflammatory and proinflammatory mediators in hepatic tissues [27, 28]. These hepatic mediators further recruited more and more inflammatory cells. In liver inflammation, various types of cells, including natural killer cells, natural killer T cells, T cells, dendritic cells, and macrophages, are recruited and activated [29]. Activated Kupffer cells produce transforming growth factor-*β* (TGF-*β*), which lead to formation of hepatic stellate cells (HSCs) into myofibroblasts. Myofibroblasts express *α*-smooth muscle actin (*α*-SMA) and produce extracellular matrix proteins (ECM), such as collagen types I, III, and IV, in the liver. Our study also showed that CCl_4_ treated rats started to develop fibrosis in liver along with the inflammatory cell infiltration. Liver fibrosis is an important step in the development of liver cirrhosis [30] which is characterized by excessive production and deposition of extracellular matrix (ECM) molecules [31, 32]. Research over the past two decades has established that hepatic stellate cells (HSC) are the primary extracellular matrix-producing cell type during hepatic fibrogenesis [31, 32].* Citrus maxima* peel powder supplementation prevented the inflammatory cell infiltration and fibrosis in CCl_4_ treated rats. This finding is in agreement with our previous report where the active constituent of citrus fruits, naringin, prevented the hepatic inflammation and fibrosis in diet induced obese rats [11]. Our observation also suggests that* Citrus maxima* peel powder supplementation prevented the iron deposition in liver of CCl_4_ treated rats. Free iron in tissue may start Fenton-like reactions which generates high level of hydroxyl free radicals and causes serious tissue damages. Several reports also suggest that iron deposition may also favor collagen deposition and fibrosis [33, 34].

Raw* Citrus maxima* is used as vegetables and in salad in Bangladesh which signifies its nontoxic characteristics. A recent study also showed that mice treated with* Citrus maxima* extract up to 2000 mg/kg dose did not cause any animal death, further confirming its nontoxic properties [35]. Moreover, our study also suggests that Control rats supplemented with* Citrus maxima* powder did not change any of the biochemical and histological indices. Thus, using this peel powder would be a safer alternative medication in liver diseases. Our results provide first evidence that* Citrus maxima* peel powder has preventive effect on CCl_4_ induced liver injury. Moreover, the presence of antioxidant compounds (caffeic acid and epicatechin) may be responsible for the effectiveness of* Citrus maxima* peel powder against liver disorders.

## Figures and Tables

**Table 1:** Contents of polyphenolic compounds in the ethanol extract of *Citrus maxima* peel extract (*n* = 5).

Polyphenolic compound	Ethanol extract of *Citrus maxima* peel extract	
	Content (mg/100 g of dry extract)	% RSD
GA	82.32	0.83
VA	13.72	0.17
CfA	240.78	1.95
SA	35.62	0.33
ECA	242.19	1.97
RH	20.35	0.21
BA	34.36	0.29

GA, gallic acid; VA, vanillic acid; CfA, caffeic acid; SA, syringic acid; ECA, (−)-epicatechin; RH, rutin hydrate; BA, benzoic acid.

**Table 2:** Effect of *Citrus maxima* peel powder on body weight, food and water intake, and organ weight of CCl_4_ treated rats.

Parameters	Control	Control + *Citrus maxima *	CCl_4_	CCl_4_ + *Citrus maxima *
Initial body weight	155.59 ± 7.60^a^	158.80 ± 1.39^a^	164.07 ± 4.70^a^	172.40 ± 3.95^a^
Final body weight	159.23 ± 10.89^a^	166.03 ± 2.90^a^	163.87 ± 6.14^a^	174.27 ± 6.09^a^
Food intake/d	18.18 ± 0.40^a^	12.61 ± 0.46^b^	12.62 ± 0.40^b^	13.76 ± 0.40^c^
Water intake/d	16.35 ± 0.37^a^	14.44 ± 0.51^b^	14.56 ± 0.48^b^	14.13 ± 0.53^c^
Liver wet weight	3.69 ± 0.11^a^	3.62 ± 0.12^a^	3.44 ± 0.07^a,c^	3.14 ± 0.07^b,c^
Kidney wet weight	0.65 ± 0.03^a^	0.63 ± 0.01^a^	0.55 ± 0.02^b^	0.56 ± 0.01^c^
Heart wet weight	0.32 ± 0.02^a^	0.29 ± 0.01^a^	0.31 ± 0.01^a^	0.33 ± 0.01^a^
Spleen wet weight	0.31 ± 0.03^a^	0.41 ± 0.01^a,c^	0.50 ± 0.02^b,c^	0.35 ± 0.02^a^

Values are presented as mean ± SEM. *N* = 7 in each group or otherwise specified. One-way ANOVA with Bonferroni tests were done as post hoc test. Values are considered significant when *p* < 0.05. a versus b, Control versus CCl_4_; b versus c, CCl_4_ versus *Citrus maxima* treatment.

**Table 3:** Effect of *Citrus maxima* peel powder on biochemical parameters in plasma and liver.

Parameters	Groups			
	Control	Control + *Citrus maxima *	CCl_4_	CCl_4 _+ *Citrus maxima *
Plasma				
AST (U/L)	25.71 ± 1.68^a^	25.71 ± 1.88^a,c^	41.20 ± 2.30^b^	31.30 ± 1.98^a,c^
ALT (U/L)	19.15 ± 0.99^a^	22.86 ± 1.41^a^	41.50 ± 1.95^b^	30.74 ± 1.93^c^
ALP (U/L)	54.40 ± 2.73^a^	61.36 ± 3.08^a,c^	81.27 ± 1.84^b^	54.40 ± 2.73^a,c^
MDA (nmol/mL)	4.31 ± 0.33^a,c^	5.49 ± 0.35^a^	7.74 ± 0.47^b^	4.31 ± 0.33^a,c^
NO (nmol/mL)	3.79 ± 0.22^a^	5.73 ± 0.26^b,c^	4.50 ± 0.65^a,c^	3.79 ± 0.22^a^
APOP (nmol/mL equivalent to chloramine-T)	111.14 ± 6.61^a^	108.29 ± 4.26^a^	242.57 ± 13.88^b^	147.00 ± 5.09^c^
Catalase (U/min)	5.96 ± 0.24^a^	8.06 ± 1.20^a^	3.67 ± 0.33^a^	7.70 ± 0.89^a,c^
GSH (nmol/mg protein)	19.71 ± 1.11^a^	20.50 ± 1.50^a^	11.14 ± 1.06^b,c^	15.29 ± 0.61^a,c^

Liver				
AST (U/L)	36.43 ± 1.43^a^	30.00 ± 2.16^a^	51.20 ± 2.52^b^	37.44 ± 1.73^a^
ALT (U/L)	31.20 ± 2.12^a^	27.00 ± 0.99^a^	50.90 ± 2.97^b^	37.60 ± 1.86^a^
ALP (U/L)	52.97 ± 2.02^a^	49.91 ± 1.53^a^	95.56 ± 3.50^b^	61.54 ± 3.60^a^
NO (nmol/mL)	13.66 ± 0.58^a,c^	12.50 ± 1.18^a,c^	22.36 ± 0.98^b^	15.36 ± 1.35^a^
MDA (nmol/mL)	13.60 ± 0.21^a^	12.20 ± 0.78^a^	27.74 ± 0.47^b^	21.17 ± 1.14^c^
APOP (nmol/mL equivalent to chloramine-T)	268.29 ± 18.04^a^	217.14 ± 8.48^a^	1236.29 ± 11.68^b^	894.00 ± 32.02^c^
Catalase (U/min)	47.39 ± 2.66^a^	43.77 ± 3.31^a^	24.67 ± 0.43^b^	34.84 ± 2.24^c^
GSH (nmol/mg protein)	39.61 ± 1.17^a^	43.36 ± 2.89^a^	21.71 ± 1.54^b^	31.00 ± 2.35^c^

Values are presented as mean ± SEM. *N* = 5–7 in each group or otherwise specified. One-way ANOVA with Bonferroni tests were done as post hoc test. Values are considered significant when *p* < 0.05. a versus b, Control versus CCl_4_; b versus c, CCl_4 _versus *Citrus maxima* treatment.
